# GPRC5D as a novel target for the treatment of multiple myeloma: a narrative review

**DOI:** 10.1038/s41408-023-00966-9

**Published:** 2024-02-02

**Authors:** Paula Rodriguez-Otero, Niels W. C. J. van de Donk, Kodandaram Pillarisetti, Ingrid Cornax, Deeksha Vishwamitra, Kathleen Gray, Brandi Hilder, Jaszianne Tolbert, Thomas Renaud, Tara Masterson, Christoph Heuck, Colleen Kane, Raluca Verona, Philippe Moreau, Nizar Bahlis, Ajai Chari

**Affiliations:** 1 Clínica Universidad de Navarra, CCUN, University of Navarra, Pamplona, Spain; 2 Amsterdam University Medical Center, Vrije Universiteit Amsterdam, Amsterdam, The Netherlands; 3 Janssen Research & Development, Spring House, PA USA; 4 Janssen Research & Development, La Jolla, CA USA; 5 Janssen Scientific Affairs, Horsham, PA USA; 6 Janssen Research & Development, Raritan, NJ USA; 7 University Hospital Hôtel-Dieu, Nantes, France; 8 Arnie Charbonneau Cancer Research Institute, University of Calgary, Calgary, Alberta Canada; 9 Icahn School of Medicine at Mount Sinai, New York, NY USA

**Keywords:** Myeloma, Drug development, Immunotherapy

## Abstract

Multiple myeloma is a genetically complex and heterogenous malignancy with a 5-year survival rate of approximately 60%. Despite advances in therapy, patients experience cycles of remission and relapse, with each successive line of therapy associated with poorer outcomes; therefore, therapies with different mechanisms of action against new myeloma antigens are needed. G protein–coupled receptor class C group 5 member D (GPRC5D) has emerged as a novel therapeutic target for the treatment of multiple myeloma. We review the biology and target validation of GPRC5D, and clinical data from early phase trials of GPRC5D-targeting bispecific antibodies, talquetamab and forimtamig, and chimeric antigen receptor T cell (CAR-T) therapies, MCARH109, OriCAR-017, and BMS-986393. In addition to adverse events (AEs) associated with T-cell–redirection therapies irrespective of target, a consistent pattern of dermatologic and oral AEs has been reported across several trials of GPRC5D-targeting bispecific antibodies, as well as rare cerebellar events with CAR-T therapy. Additional studies are needed to understand the underlying mechanisms involved in the development of skin- and oral-related toxicities. We review the strategies that have been used to manage these GPRC5D-related toxicities. Preliminary efficacy data showed overall response rates for GPRC5D-targeting T-cell–redirecting therapies were ≥64%; most responders achieved a very good partial response or better. Pharmacokinetics/pharmacodynamics showed that these therapies led to cytokine release and T-cell activation. In conclusion, results from early phase trials of GPRC5D-targeting T-cell–redirecting agents have shown promising efficacy and manageable safety profiles, including lower infection rates compared with B-cell maturation antigen- and Fc receptor-like protein 5-targeting bispecific antibodies. Further clinical trials, including those investigating GPRC5D-targeting T-cell–redirecting agents in combination with other anti-myeloma therapies and with different treatment modalities, may help to elucidate the future optimal treatment regimen and sequence for patients with multiple myeloma and improve survival outcomes.

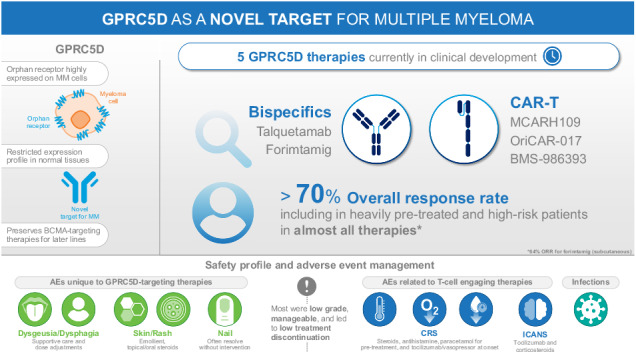

## Introduction

Multiple myeloma (MM) is a genetically complex and heterogenous malignancy that is associated with substantial morbidity and mortality [1–3]. It accounts for 15%–20% of all hematologic malignancies and has a 5-year survival rate of 60% [4–6]. MM can affect multiple organ systems, leading to fatigue, pain, breathlessness, and mobility issues, as well as neurological effects (including numbness/tingling), renal impairment, bone disease, blood disorders, and frequent infections [7–13]. The symptom burden is associated with poor health-related quality of life, including depression, anxiety, poor function, and lower social satisfaction [14].

Despite advances in therapy, including B-cell maturation antigen (BCMA)-targeting antibody-drug conjugates, bispecific antibodies, and chimeric antigen receptor T-cell (CAR-T) therapies, patients experience cycles of remission and relapse; with each new line of therapy (LOT), the risk of discontinuation increases due to poor efficacy, accumulating toxicities, and increasingly prevalent comorbidities [15–17]. New therapies with different mechanisms of action against new targets are needed across LOT to improve patient outcomes. G protein–coupled receptor class C group 5 member D (GPRC5D) is a novel therapeutic target for the treatment of MM. Here, we describe the biology and target validation of GPRC5D and review data from GPRC5D-targeting T-cell–redirecting agents in clinical development.

## Biology of GPRC5D

**Fig. 1:**
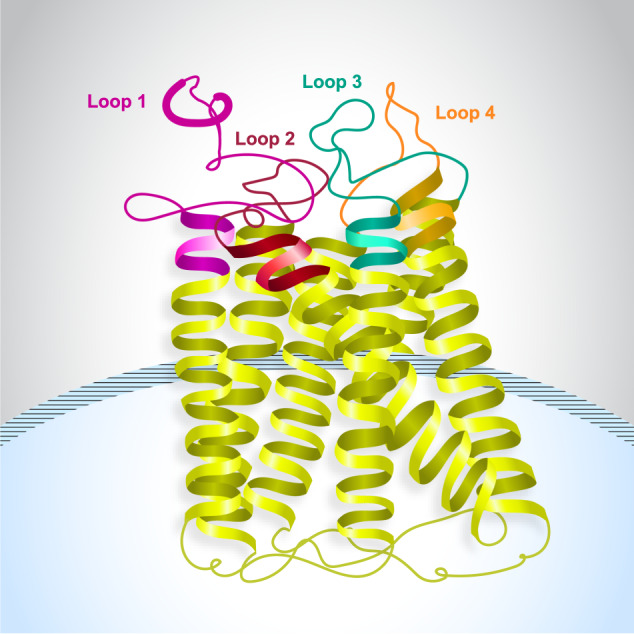
Structure of G protein–coupled receptor class C group 5 member D (GPRC5D). Graphical representation of GPRC5D showing the transmembrane region, which crosses the lipid bilayer seven times, and short extracellular N-terminal domain.

GPRC5D, located on human chromosome 12p13, is an orphan G-protein coupled receptor, meaning that its ligand is unknown (Fig. 1) and due to the lack of in vivo models, its signaling mechanism and function in normal tissue and MM are not yet established [18–21]. Due to the short extracellular domains, the exposed epitopes of GPRC5D for T-cell–redirecting agents are likely closer to the plasma membrane, facilitating tighter immunological synapses between T cells and target cells that may drive greater cytotoxicity [20, 22]. As a seven-pass transmembrane receptor protein with a short extracellular N-terminal domain, GPRC5D is unlikely to be shed from target cells into the serum, which may reduce the risk of decreased efficacy related to target antigen shedding [20, 23].

## GPRC5D expression in normal tissue

**Fig. 2:**
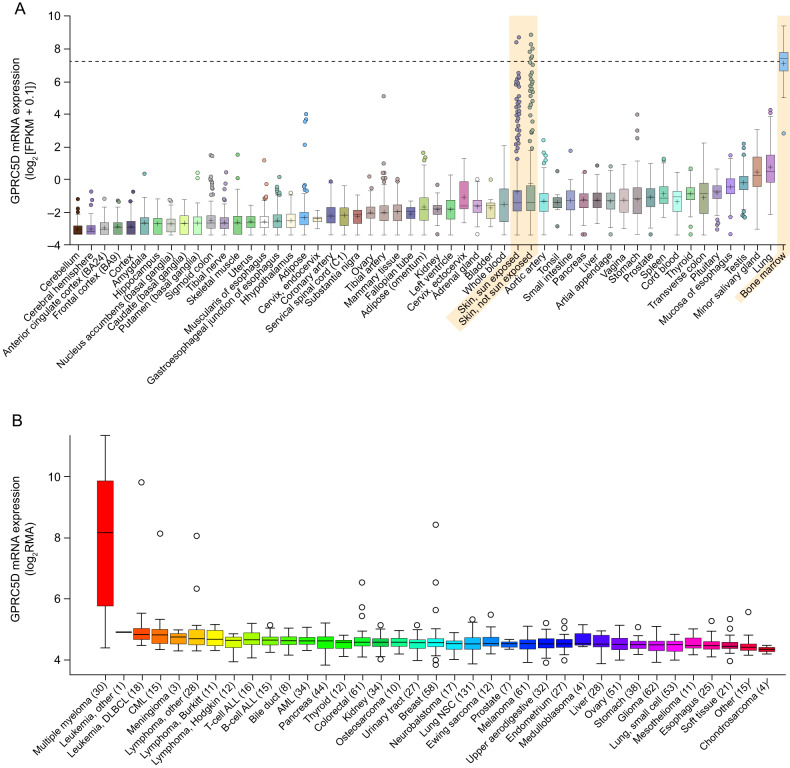
Expressionof G protein–coupled receptor class C group 5 member D (GPRC5D) in healthy tissues and in multiplemyeloma (MM) versus other cancer types. The first panel (**A**) shows mRNA expression levels of GPRC5D across a range of healthy tissues. The second panel (**B**) shows mRNA expression levels of GPRC5D in multiple myeloma compared with expression levels observed across a range of hematological and solid tumor malignancies. ALL acute lymphoblastic leukemia, AML acute myeloid leukemia, CML chronic myelogenous leukemia, DLBCL diffuse large B- cellly mphoma, NSC non-small cell. Reproduced from Smith EL, et al. GPRC5D is a target for the immunotherapy of multiple myeloma with rationally designed CAR T cells. Sci Transl Med. 2019;11(485):eaau7746.

Although there is a relative paucity of high-quality immunohistochemistry reagents for the detection of GPRC5D protein in normal (non-diseased) and tumor tissues, GPRC5D expression profiling can be accomplished by augmenting immunohistochemical data with complementary assays, such as in situ hybridization (ISH), RNA-sequencing, and flow cytometry [19, 20, 24, 25]. GPRC5D protein has been detected in immune cells and in epithelial structures of the skin and tongue [24]. In the immune cell compartment, GPRC5D protein is predominantly expressed in cells with a plasma cell phenotype [20, 21, 26] and has little to no expression in normal B cells, T cells, natural killer cells, monocytes, granulocytes, and bone marrow progenitors [21, 25–27], unlike CD38 and BCMA, which have a broader expression profile. In epithelium, GPRC5D expression has been detected in hair follicles (during the mid- and late-anagen and catagen phases only), in eccrine glands, in the skin (hair follicle–specific keratin), and at the base of filiform papillae on the tongue [19, 21, 24, 25, 28]. Expression of GPRC5D is suspected to occur in the nailbed in humans because GPRC5D messenger RNA (mRNA) expression has been reported in the nailbeds of mice [19]. GPRC5D mRNA expression in other healthy tissues is shown in Fig. 2A, with no GPRC5D expression detected in the human testis by immunohistochemistry (IHC) [25].

Low levels of GPRC5D mRNA have been detected in the motor neurons of the inferior olivary nucleus of the brainstem in the central nervous system by ISH, but without correlative protein detection by IHC [24, 29]. No GPRC5D mRNA or protein expression has been detected in the cerebral cortex, basal ganglia, midbrain, or cerebellum by ISH or IHC [24]. At present, no data are available to describe potential racial differences in GPRC5D expression.

## GPRC5D as target for MM treatment

Successful T-cell–redirection therapy is dependent on eliminating malignant cells expressing a target that has minimal to no expression in other tissues [20]. GPRC5D is highly expressed in MM cells and is abundant in the bone marrow from patients with MM and smoldering MM [20, 25, 27, 30]. Additionally, GPRC5D mRNA expression is also higher in MM cells versus other hematologic cancers (Fig. 2B) [25]. The selective expression in MM cells suggests that GPRC5D is an ideal target for immune effector cell–mediated therapy to treat MM [25, 26]. While GPRC5D and BCMA have similar expression on CD138+ cells, the expression patterns are independent of each other, offering distinct clinical targets [25]. GPRC5D expression is also unaffected by BCMA loss, which has been associated with disease relapse following treatment with BCMA-targeting therapies and may support combining GPRC5D-targeting and BCMA-targeting T-cell–redirecting agents to address the heterogeneity of MM [20, 31].

Increased GPRC5D mRNA expression from bone marrow or MM cells is associated with a high number of genetic aberrations and high-risk disease [21, 26, 27]; however, this may be due to the proportions of malignant plasma cells, which have elevated levels of GPRC5D expression, in bone marrow samples. GPRC5D is also associated with International Staging System (ISS) stage and β_2_-macroglobulin [27]. Increased GPRC5D mRNA expression in bone marrow has been associated with poor prognosis and disease outcomes, including shorter progression-free survival and overall survival [25, 27]. However, many of these observations may be due to an overall increase in tumor burden and a higher proportion of features associated with high-risk disease, as a causative relationship between GPRC5D and malignant plasma cell transformation or high-risk disease has not been demonstrated. Furthermore, genome and RNA sequencing in patients with MM showed that a significant number of patients have aberrations in genes encoding immunotherapy targets, including GPRC5D. However, while 15% of patients had heterozygous deletion of GPRC5D, these aberrations did not impact gene expression [32]. A recent analysis reported four cases of relapse with biallelic mutations of GPRC5D following GPRC5D-targeting bispecific antibody therapy, including two cases with convergent evolution where multiple subclones lost GPRC5D through somatic events [33]. GPRC5D loss has also been observed with GPRC5D-targeting CAR-T therapy [29]. Biallelic genetic inactivation of GPRC5D has also been described elsewhere in the literature as a mechanism of resistance, along with long-range epigenetic silencing of its promoter and enhancer regions [34].

## GPRC5D-targeted therapies in clinical development for the treatment of MM

Two GPRC5D×CD3 bispecific antibodies and 3 GPRC5D-targeted CAR-T therapies are in development for the treatment of patients with MM (Table 1).

**Table 1:** Completed and ongoing clinical trials of GPRC5D-targeting agents.

Trial name (ClinicalTrials.gov identifier)	Phase	Intervention	Patient population	Primary endpoint
Talquetamab				
MonumenTAL-1 (NCT03399799/ NCT04634552)	Phase 1/2	Talquetamab monotherapy	Phase 1: Patients with RRMM who had progressed on or were intolerant to established therapies Phase 2: Patients with RRMM who had previously received ≥3 prior lines of therapy, including a PI, an IMiD, and an anti-CD38 antibody	Phase 1: Safety and dose-limiting toxicities Phase 2: ORR
MonumenTAL-2 (NCT05050097)	Phase 1b	Talquetamab + carfilzomib, daratumumab, lenalidomide, or pomalidomide	Patients with RRMM who have a ECOG performance status of 0/1	Safety and dose-limiting toxicities
TRIMM-2 (NCT04108195)	Phase 1b	Talquetamab or teclistamab + daratumumab ± pomalidomide	Patients with RRMM who have received ≥3 prior lines of therapy, including a PI, and an IMiD, or were double refractory to a PI and an IMiD, and had not received anti-CD38 therapy within 90 days	Safety and dose-limiting toxicities
TRIMM-3 (NCT05338775)	Phase 1b	Talquetamab or teclistamab + a PD-1 inhibitor	Patients with RRMM who are not candidates for available therapy with established clinical benefit	Safety and dose-limiting toxicities
RedirecTT-1 (NCT04586426)	Phase 1b/2	Talquetamab + teclistamab ± daratumumab	Parts 1 & 2: Patients could not tolerate or have disease that is relapsed or refractory to established therapies, including the last line of therapy. Part 3: Relapsed or refractory disease, and exposed to a PI, an IMiD, and an anti-CD38 antibody; no prior BCMA-targeted bispecific antibody or prior GPRC5D targeted therapy allowed	Safety and dose-limiting toxicities
Forimtamig				
NCT04557150	Phase 1	Forimtamig monotherapy	Patients with RRMM who have previously received therapy with a PI and an IMiD and are intolerant to or have no other option for standard-of-care treatment per the investigator	Safety and dose-limiting toxicities
MCARH109				
NCT04555551	Phase 1	MCARH109	Patients with RRMM who have previously received ≥3 prior lines of therapy, including a PI, an IMiD, and an anti-CD38 antibody therapy	Maximum tolerated dose
NCT05431608	Phase 1	MCARH109 + MCARH125	Patients with RRMM who have previously received ≥3 prior lines of therapy, including a PI, an IMiD, and an anti-CD38 antibody therapy	Maximum tolerated dose
OriCAR-017				
POLARIS (NCT05016778)	Phase 1	OriCAR-017	Patients who have received ≥3 lines of therapy, including chemotherapy, a PI, and an immunosuppressive agent, and have failed treatment or have progressed or recurred during the last treatment or within 6 months after the end of treatment	Safety and dose-limiting toxicities
BMS-986393				
NCT04674813	Phase 1	BMS-986393	Patients who have received ≥3 lines of therapy including a PI, an IMiD, and an anti-CD38 antibody therapy and, unless ineligible, prior stem cell transplant. Prior BCMA-directed and CAR-T therapies are allowed	Safety, dose-limiting toxicities, and maximum tolerated dose

*BCMA* B-cell maturation antigen, *CAR-T* chimeric antigen receptor T cell, *ECOG* Eastern Cooperative Oncology Group, *IMiD* immunomodulatory drug, *ORR* overall response rate, *PI* proteasome inhibitor, *RRMM* relapsed/refractory multiple myeloma.

## T-cell–redirecting bispecific antibodies

Bispecific antibodies have been engineered to overcome some of the limitations of conventional monoclonal antibodies and enhance therapeutic efficacy [35]. There are two antigen-binding sites on T-cell–redirecting bispecific antibodies: CD3 on the surface of T cells and a target antigen that is expressed on tumor cells [35]. Binding to both the T-cell surface receptor and tumor-specific antigen brings the T cells into proximity with tumor cells, leading to synapse formation and induction of T-cell activation and subsequent killing of malignant cells [36]. T-cell–redirecting antibodies may overcome some forms of tumor-mediated evasion from the immune system because they do not require antigen presentation by major histocompatibility complex molecules, which are often downregulated or lost in tumor cells, to carry out effector functions [36].

**Fig. 3:**
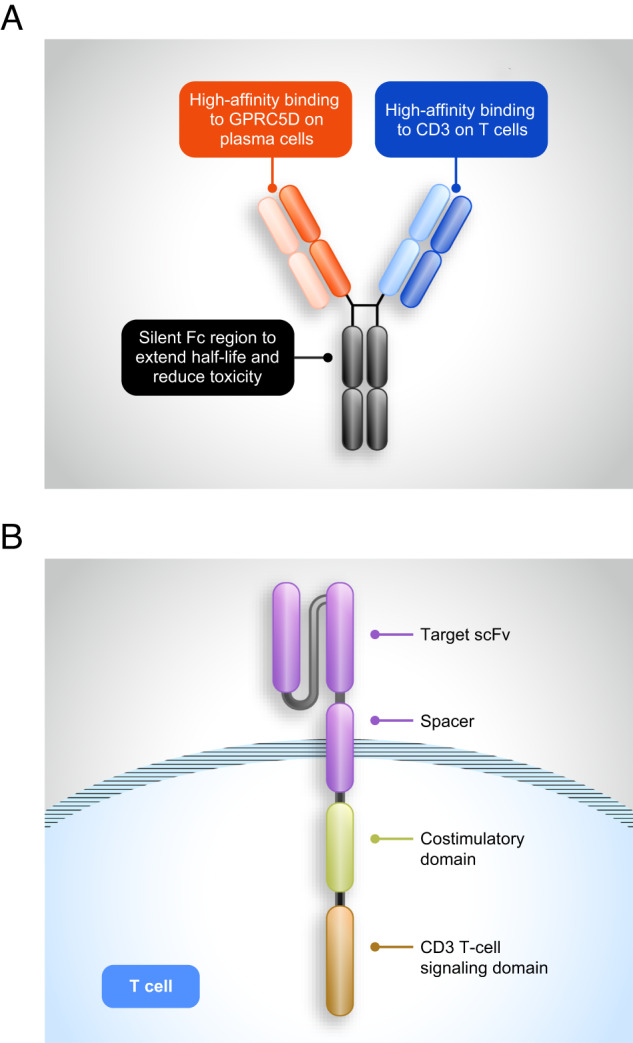
Structure of bispecific antibodies and GPRC5D-targeting CAR-T therapies. The first panel (**A**) shows a generalized structure ofGPRC5D-targeting bispecific antibodies with the Fc region and CD3 and antigen-binding regions depicted. The second panel (**B**) shows the structure of CAR-T therapies with the target scFv and spacer regions and costimulatory and CD3 signaling domains depicted.

Bispecific antibodies can have different constructs, including single-chain variable fragments (scFv), which do not include an Fc domain, and full-size antibody constructs generated through methods, such as Fab-arm exchange [37, 38]. Some of the scFv-based antibodies (e.g., BiTEs^®^) are relatively small molecules (~25 kDa) with an antigen-binding site generated by fusing immunoglobulin G (IgG) heavy- and light-chain variable regions through a flexible polypeptide linker [36, 39]. The lack of an Fc domain is thought to prevent activation of off-target effector functions, including antibody-dependent cell-mediated cytotoxicity, complement-dependent cytotoxicity, and antibody-dependent cellular phagocytosis; however, modifications may be needed to extend the half-life in vivo [38]. In full-length IgG-like antibodies, the IgG portion resembles a natural IgG molecule, with two antigen-binding regions joined via an Fc stem (Fig. 3A) [38]. Inclusion of the Fc domain contributes to a more stable antibody with longer serum half-life than scFv antibodies [38, 40]. With an Fc domain, full-size antibodies may retain effector functions; however, the Fc domain can be functionally “silenced” to prevent nonspecific effector functions. Talquetamab and forimtamig both have silenced Fc domains.

### Structure, mechanism of action, and preclinical evidence of T-cell–redirecting bispecific antibodies

#### Talquetamab

Talquetamab is an off-the-shelf, full-sized, humanized, T-cell–redirecting IgG4 bispecific antibody targeting both GPRC5D and CD3 receptors [20, 21, 41, 42]. Talquetamab is the first GPRC5D-targeting agent approved for the treatment of patients with triple-class exposed relapsed/refractory MM [43, 44].

Preclinically, talquetamab promoted efficient T-cell activation and T-cell–mediated cytotoxicity across three different GPRC5D positive (+) cell lines (H929, MM.1 R, and OPM2), with no effect on GPRC5D-negative cell lines [20]. Additionally, talquetamab induced cell lysis in primary MM cells from bone marrow aspirates in newly diagnosed, daratumumab-naive relapsed/refractory, and daratumumab-refractory patients with MM [21]. This cell lysis was induced regardless of resistance to prior therapies or the presence of high-risk cytogenetic abnormalities [21].

#### Forimtamig

Forimtamig is a T-cell–redirecting bispecific antibody that binds to CD3 on T cells and GPRC5D on plasma cells [28, 45]. It has a unique 2:1 (GPRC5D:CD3) configuration for hypothetical increased potency versus a 1:1 configuration [28, 45, 46]. Dual binding induces T-cell–directed killing of plasma cells [28, 45].

Preclinically, forimtamig exhibited cytotoxicity against all GPRC5D + MM cell lines against which it was tested [47]. In an autologous ex vivo model of MM samples using total bone marrow aspirates from newly diagnosed patients with MM, forimtamig increased cytotoxicity potency. Forimtamig also inhibited growth of established NCI-H929 xenograft tumors in humanized mice after repeat dosing and eradicated tumors at doses of 0.1–10.0 mg/kg.

## CAR-T therapies

CAR-T cells have been described as a breakthrough in cancer immunotherapy, revolutionizing the treatment of B-cell malignancies and improving prognosis in patients with relapsed/refractory MM [48–50]. CAR-T therapy uses a patient’s own T cells, engineered to target tumor cells through expression of CARs comprising an scFv, a CD3ζ T-cell receptor molecule, and a costimulatory domain (usually CD28 or 4-1BB) (Fig. 3B) [51].

CAR-T therapies are typically administered as a single dose [51]. GPRC5D-targeting CAR-T therapies in clinical development are MCARH109, OriCAR-017, and BMS-986393 (Table 1).

### Structure, mechanism of action, and preclinical evidence of CAR-T therapies

#### MCARH109, OriCAR-017, and BMS-986393

MCARH109 is a second-generation CAR-T therapy with a human B-cell–derived anti-GPRC5D single-chain variable fragment, a 4-1BB costimulatory domain, and CD3 signaling domain [29]. OriCAR-017 is an autologous, second-generation, GPRC5D-targeting CAR-T therapy [52, 53]. The proprietary signal activation domain element Ori is hypothesized to improve CAR-T expansion and durability [53]. BMS-986393 is a GPRC5D-targeting autologous CAR-T therapy that has the same structure as MCARH109 [54].

The in vitro and in vivo antitumor activity of GPRC5D CAR-T cells has been shown in a preclinical study of a CAR-T therapy incorporating a human-derived GPRC5D-targeted scFv clone 109 (GPRC5D[109]) using MM cell lines [25]. GPRC5D(109), which is a precursor to MCARH109 [29], eradicated primary MM cells obtained via bone marrow aspirate and other MM cell lines with a range of GPRC5D mRNA expression profiles [25].

### Clinical data

In total, ten clinical trials of GPRC5D-targeting T-cell–redirecting agents are either completed or ongoing (Table 1). Of these, five trials have published results, which are summarized below: MonumenTAL-1 (NCT03399799/NCT04634552), NCT04557150, NCT04555551, POLARIS (NCT05016778), and NCT04674813 (Tables 1, 2).

**Table 2:** Key trial design and patient baseline characteristics from GPRC5D-targeting trials.

	Talquetamab^a^	Forimtamig^c^	MCARH109	OriCAR-017	BMS-986393
Construct	GPRC5D×CD3 bispecific antibody	GPRC5D×CD3 bispecific antibody	GPRC5D-targeted CAR-T	GPRC5D-targeted CAR-T	GPRC5D-targeted CAR-T
Trial name (ClinicalTrials.gov identifier)	MonumenTAL-1 (NCT03399799/ NCT04634552)	NCT04557150	NCT04555551	NCT05016778	NCT04674813
Phase	Phase 1/2	Phase 1	Phase 1	Phase 1	Phase 1
Population size	*N* = 288 (*n* = 143 treated with 0.4 mg/kg QW SC; *n* = 145 treated with 0.8 mg/kg Q2W SC)^b^	*N* = 108 (*n* = 51 treated in IV cohort; *n* = 57 treated in SC cohort)	*N* = 17	*N* = 10	*N* = 33
Dosing	Phase 1: 0.5–180 µg/kg IV and 5–1600 µg/kg SC Phase 2: 0.4 mg/kg QW SC or 0.8 mg/kg Q2W SC (RP2D)	18−10,000 µg IV Q2W, aside from Q3W at the 18/162/7200 μg IV dosing level 1200−7200 µg SC Q2W, aside from Q3W at the 30/150/4800 μg and 30/300/ 7200 μg SC dosing level	1 infusion of 25 × 10^6^, 50 × 10^6^, 150 × 10^6^, or 450 × 10^6^ CAR-T cells	1 infusion of 1 × 10^6^/kg, 3 × 10^6^/kg, or 6 × 10^6^/kg CAR-T cells	1 infusion of 25 × 10^6^, 75 × 10^6^, 150 × 10^6^, 300 × 10^6^, or 450 × 10^6^ CAR-T cells
Last presented Data cut-off	ASH 2022 May 16, 2022 (safety) September 12, 2022 (efficacy)	ASH 2022 October 21, 2022	*N Engl J Med* 2022 June 16, 2021	*Lancet Haematol* 2023 June 30, 2022	ASH 2022 September 7, 2022
Dosing schedule Step-up	SC QW/Q2W 2/3 step-up doses	IV/SC Q2W, with noted exceptions 2 step-up doses	Bridging therapy after apheresis was allowed if it was stopped ≥2 weeks before lymphodepleting chemotherapy. Patients received lymphodepleting chemotherapy with fludarabine (30 mg/m^2^/d) and cyclophosphamide (300 mg/m^2^/d) for 3 days. Two days after lymphodepletion, patients received a single MCARH109 infusion	Prior BCMA and bridging treatments were allowed. Patients received lymphodepleting chemotherapy with fludarabine (30 mg/m^2^/d) and cyclophosphamide (300 mg/m^2^/d) for 3 days followed by a single infusion of OriCAR-017	Prior BCMA-directed therapies were allowed, including CAR-T therapy. Patients received lymphodepleting chemotherapy with fludarabine (30 mg/m^2^/d) and cyclophosphamide (300 mg/m^2^/d) for 3 days followed by a single infusion of BMS-986393
Median follow-up (range)	14.9 mo (0.5–29.0)/ 8.6 mo (0.2–22.5)	11.6 mo (0.5–20.6)/8.0 mo (1.1–15.0)	10.1 mo (8.5–NE)	238 days (182–307^d^)	5.8 mo (1.0–15.5)
Median prior lines (range)	5 (2–13)/5 (2–17)	5 (2–15)/4 (2–14)	6 (4–14)	5.5 (4–10^d^)	4 (3–13)
Triple-class refractory	106 (74%)/100 (69%)	31 (62%)/41 (72%)	16 (94%)	N/R	N/R
Penta-drug refractory	42 (29%)/34 (23%)	18 (36%)/24 (42%)	N/R	N/R	N/R

*BCMA* B-cell maturation antigen, *CAR-T* chimeric antigen receptor T cell, *GPRC5D* G protein–coupled receptor family C group 5 member D, *IV* intravenous, *mo* months, *NE* not estimable, *N/R* not reported, *Q3W* every 3 weeks, *Q2W* every other week, *QW* weekly, *RP2D* recommended phase 2 dose, *RRMM* relapsed/refractory multiple myeloma, *SC* subcutaneous.

^a^Data presented are for the 0.4 mg/kg and 0.8 mg/kg groups.

^b^All patients were treated with the R2PD.

^c^Data presented are for the IV and SC cohorts.

^d^Data are interquartile ranges.

#### Safety

##### Talquetamab

In MonumenTAL-1, patients had a median of five prior LOT, and 29% and 23% of patients treated with the subcutaneous (SC) recommended phase 2 doses (RP2Ds) of 0.4 mg/kg weekly (QW; [*n* = 143]) and 0.8 mg/kg biweekly (Q2W; [*n* = 145]), respectively, were penta-drug refractory [42]. In the 0.4 mg/kg QW and 0.8 mg/kg Q2W cohorts, 31% and 29% had high-risk cytogenetics, respectively [42].

A summary of safety results across trials of GPRC5D-targeting T-cell–redirecting agents, including from the MonumenTAL-1 trial [42], is shown in Table 3. In MonumenTAL-1, there were low rates of discontinuation due to adverse events (AEs) observed with 0.4 mg/kg QW and 0.8 mg/kg Q2W dosing (5% and 6%, respectively); 8% and 14% had dose delays due to AEs, and 15% and 6% had dose reductions due to AEs. No patients died due to drug-related AEs [42].

**Table 3:** Safety summary across GPRC5D-targeting agents.

	Talquetamab				Forimtamig^b^				MCARH109^c^ (*N* = 17)		OriCAR-017^d^ (*N* = 10)		BMS-986393^e^ (*N* = 33)	
	0.4 mg/kg SC QW^a^ (*n* = 143)		0.8 mg/kg SC Q2W (*n* = 145)		IV cohort (*n* = 51)		SC cohort (n = 57)							
AEs of interest, *n* (%)	Any grade	Grade 3/4	Any grade	Grade 3/4	Any grade	Grade ≥3	Any grade	Grade ≥3	Any grade	Grade 3/4	Any grade	Grade 3/4	Any grade	Grade 3/4
Anemia	64 (45)	45 (32)	57 (39)	36 (25)	17 (33)	8 (16)	28 (49)	22 (39)	15 (88)	7 (41)	8 (80)	7 (70)	12 (36)	7 (21)
Neutropenia	49 (34)	44 (31)	41 (28)	32 (22)	12 (24)	6 (12)	10 (18)	9 (16)	17 (100)	17 (100)	10 (100)	10 (100)	22 (67)	20 (61)
Lymphopenia	40 (28)	37 (26)	38 (26)	37 (26)	N/R	N/R	N/R	N/R	17 (100)	17 (100)	3 (30)	2 (20)	N/R	N/R
Thrombocytopenia	39 (27)	29 (20)	39 (27)	24 (17)	16 (31)	7 (14)	15 (26)	11 (19)	15 (88)	11 (65)	9 (90)	9 (90)	13 (39)	7 (21)
CRS	113 (79)	3 (2)	105 (72)	1 (1)	42 (82)	1 (2)	45 (79)	1 (2)	15 (88)	1 (6)	10 (100)	0	21 (64)	2 (6)
ICANS^f^	13 (11)	2 (2)	11 (10)	2 (2)	5 (10)	1 (2)	7 (12)	2 (4)	1 (6)	1 (6)	0	0	2 (6)	0
Skin-related AEs^g,h,i,j,k^	80 (56)	0	98 (68)	1 (1)	40 (78)	6 (12)	49 (86)	13 (23)	1 (6)	0	3 (30)	0	10 (30)	0
Hair and nail changes	N/R	N/R	N/R	N/R	12 (24)	0	16 (28)	0	N/R	N/R	N/R	N/R	N/R	N/R
Nail-related AEs^l,m,n^	74 (52)	0	63 (43)	0	N/R	N/R	N/R	N/R	11 (65)	0	3 (30)	0	3 (9)	0
Dysgeusia^o^	69 (48)	N/A	67 (46)	N/A	N/R	N/R	N/R	N/R	2 (12)	N/A	N/R	N/R	5 (15)	N/A
Dysphagia	34 (24)	0	33 (23)	3 (2)	N/R	N/R	N/R	N/R	N/R	N/R	N/R	N/R	1 (3)	0
Dry mouth	36 (25)	0	53 (37)	0	N/R	N/R	N/R	N/R	1 (6)	0 (0)	N/R	N/R	N/R	N/R
Mucosal toxicity^p^	N/R	N/R	N/R	N/R	37 (73)	0	44 (77)	3 (5)	N/R	N/R	N/R	N/R	N/R	N/R
Rash-related AEs^q^	56 (39)	2 (1)	39 (27)	8 (6)	N/R	N/R	N/R	N/R	3 (18)	0	N/R	N/R	N/R	N/R
Infections	82 (57)	24 (17)	73 (50)	17 (12)	31 (61)	11 (22)	26 (46)	15 (26)	3 (18)	2 (12)	N/R	N/R	N/R	N/R

Data cut-off: May 16, 2022 (talquetamab); October 21, 2022 (forimtamig); June 30, 2022 (OriCAR-017); patients were enrolled between September 15, 2020, and June 16, 2021 (MCARH109); September 7, 2022 (BMS-986393).

*AE* adverse event, *CAR-T* chimeric antigen receptor T cell, *CRS* cytokine release syndrome, *GI* gastrointestinal, *GPRC5D* G protein–coupled receptor family C group 5 member D, *ICANS* immune effector cell–associated neurotoxicity syndrome, *IV* intravenous, *N/A* not applicable, *N/R* not reported, *Q2W* every other week, *QW* weekly, *RP2D* recommended phase 2 dose, *SC* subcutaneous.

^a^In phase 1, 0.405 mg/kg SC QW was one of the two RP2Ds; 0.4 mg/kg SC QW was selected as final dosing concentration in phase 2 for operational convenience.

^b^Data presented are for all IV and SC doses combined.

^c^Three patients received 25 × 10^6^ CAR-T cells and 50 × 10^6^ CAR-T cells, six patients received 150 × 10^6^ CAR-T cells, and five patients received 450 × 10^6^ CAR-T cells.

^d^Three patients each received 1 × 10^6^ CAR-T cells per kg, 3 × 10^6^ CAR-T cells per kg, and 6 × 10^6^ CAR-T cells per kg, respectively, in the dose-escalation phase. In the dose-expansion phase, one additional patient received the RP2D of 3 × 10^6^ CAR-T cells per kg.

^e^Thirty-three patients received doses of BMS-986393 at 25 (*n* = 6), 75 (*n* = 9), 150 (*n* = 11), 300 (*n* = 6), and 450 (*n* = 1) × 10^6^ CAR-T cells.

^f^In the ICANS analysis, 122 and 109 patients received talquetamab QW and Q2W, respectively.

^g^Includes skin exfoliation, dry skin, pruritus, and palmar-plantar erythrodysesthesia syndrome with talquetamab treatment.

^h^Includes rash, skin exfoliation erythema, skin toxicity, dermatitis, dermatitis exfoliative, toxic skin eruption, eczema, erythematous rash, macular rash, and maculopapular rash with forimtamig treatment.

^i^Includes pruritus with MCARH109 treatment.

^j^Includes pruritus and dry skin with OriCAR-017 treatment.

^k^Includes pruritus, maculopapular rash, pain from skin, erythema, and vesicular rash with BMS-986393 treatment.

^l^Includes nail discoloration, nail disorder, onycholysis, onychomadesis, onychoclasis, nail dystrophy, nail toxicity, and nail ridging with talquetamab treatment.

^m^Includes nail disorder with OriCAR-017 treatment.

^n^Includes nail bed disorder, nail discoloration, and nail disorder with BMS-986393 treatment.

^o^Dysgeusia has a maximum severity of grade 2 per Common Terminology Criteria for Adverse Events guidelines.

^p^Includes dysgeusia, dry mouth, ageusia, stomatitis, salivary hypersecretion, mucosal inflammation, anosmia, dry lip, lip edema, mucosal dryness, mucosal toxicity, and oral paresthesia with forimtamig treatment.

^q^Includes rash, maculopapular rash, erythematous rash, and erythema in patients treated with talquetamab and rash in patients who received MCARH109.

The most common grade 3/4 AEs were hematologic: anemia, neutropenia, lymphopenia, and thrombocytopenia [42]. Cytopenias were mostly confined to the first few cycles. The most common non-hematologic AEs were cytokine release syndrome (CRS), skin-related events, nail-related events, and dysgeusia. Dermatologic AEs were mostly grade 1/2. Grade 3/4 rashes occurred in two patients (1%) at 0.4 mg/kg QW and eight patients (6%) at 0.8 mg/kg Q2W, and one patient (1%) had a grade 3/4 skin-related AE in the 0.8 mg/kg Q2W cohort. Oral-related AEs included dysgeusia, dry mouth, and dysphagia, which were mostly grade 1/2, except for three (2%) patients in the 0.8 mg/kg Q2W cohort who had grade 3 dysphagia. Any-grade weight loss was experienced by 40% of patients in the 0.4 mg/kg QW cohort and 32% of patients in the 0.8 mg/kg Q2W cohort [42].

CRS events were mostly grade 1/2 and were largely confined to step-up doses and the first full dose. Median time to onset of CRS was 48 h, with a median duration of 48 h in both cohorts [42]. In total, 74% and 69% of patients in each cohort, respectively, received supportive measures for CRS, most commonly tocilizumab (35% and 37%, respectively). Immune effector cell–associated neurotoxicity syndrome (ICANS), which was assessed in phase 2 only, occurred in 11% and 10% of patients treated with each dose, respectively, and were mostly grade 1/2. Across both doses, 7%–8% of patients received supportive measures for ICANS, including tocilizumab and corticosteroids. Most patients (86% and 79% in each cohort, respectively) recovered from ICANS [42].

Grade 3/4 infections occurred in 17% of patients treated with 0.4 mg/kg QW and in 12% of patients treated with 0.8 mg/kg Q2W [42]. Opportunistic infections occurred in 4% of patients in the 0.4 mg/kg QW cohort and in 3% patients in the 0.8 mg/kg Q2W cohort. COVID-19 occurred in 9% of patients in the 0.4 mg/kg QW cohort and 11% in the 0.8 mg/kg Q2W cohort, which led to death in two patients. Intravenous (IV) immunoglobulin support was low with 0.4 mg/kg QW (13%) and 0.8 mg/kg Q2W (10%) dosing [42].

##### Forimtamig

In the phase 1 trial of forimtamig, 51 patients received IV forimtamig 18–10,000 µg and 57 patients received SC forimtamig 1200−7200 µg, preceded by step-up doses [45]. Patients had a median of five prior LOT in the IV cohort and four prior LOT in the SC cohort; 36% and 42% of patients were penta-drug refractory in each cohort, respectively. High-risk cytogenetics were observed in 47% of patients in both cohorts [45].

In the IV and SC cohorts, respectively, six (12%) and two (4%) patients had AEs leading to dose reductions; three (6%) and five (9%) patients had an AE leading to forimtamig discontinuation [45]. In the IV cohort, three patients died due to AEs (*Escherichia coli* sepsis, malignant neoplasm, and pneumonia aspiration; none considered related to treatment), and two patients died due to AEs in the SC cohort (COVID-19, considered unrelated to treatment, and acute respiratory failure, considered related to treatment). CRS was a commonly reported AE, was generally low grade, confined to the first cycle, and had a median time to onset of 5 h in the IV cohort and 24 h in the SC cohort; most CRS events resolved (98%). Twenty-seven (53%) and 14 (26%) patients required corticosteroids in the IV and SC cohorts, respectively, to manage CRS; 20 (39%) and 14 (26%) were treated with tocilizumab, respectively. Single vasopressors were administered to one patient in each cohort. Central nervous system toxicity consistent with ICANS was observed in five patients (10%) in the IV cohort (headache [*n* = 3], including one grade ≥3 event; confusion [*n* = 1]; and muscular weakness [*n* = 2]) and seven (12%) patients in the SC cohort (headache [*n* = 1]; confusion [*n* = 2]; insomnia [*n* = 2]; encephalopathy [*n* = 1]; ICANS [*n* = 1], which was grade ≥3; and syncope [*n* = 1], which was grade ≥3). Hematologic AEs were common in both cohorts (Table 3). AEs related to GPRC5D expression on non-myeloma cells were mostly grade 1/2, including skin-related AEs, mucosal AEs, and hair and nail changes [45].

##### MCARH109

The phase 1 trial of MCARH109 enrolled 17 patients, many of whom with high-risk features [29]. The median number of prior LOT was six, and ten (59%) patients received prior BCMA-targeting therapies, including eight (47%) who received prior BCMA CAR-T therapy. In total, 76% had high-risk cytogenetics [29].

All patients had ≥1 AE that emerged or worsened after MCARH109 infusion (Table 3) [29]. Dose-limiting toxicities were observed in patients who received the 450 × 10^6^ CAR-T cells dose (one patient had three grade 4 events: CRS, ICANS, and macrophage activation syndrome; two patients had grade 3 cerebellar disorder possibly related to MCARH109) [29]. Grade 3/4 neutropenia, thrombocytopenia, and anemia occurred in 100%, 65%, and 41% of patients, respectively. Non-hematologic grade 3/4 events were uncommon. Two patients (12%) had grade 3 infections (bacterial infection and parvovirus infection).

Time-limited on-target, off-tumor toxic effects related to GPRC5D expression in the skin and keratinized tissue manifested as grade 1 nail changes, including nail loss in 11 patients (65%) (at all dose levels), grade 1 rashes, and grade 1 dysgeusia or dry mouth. The median time from MCARH109 infusion to nail changes was 3.3 months, and nail changes resolved in 10/11 patients (91%) without intervention. Rash was treated with topical glucocorticoids or observation alone, and symptoms resolved; dysgeusia resolved in both patients without intervention [29].

##### OriCAR-017

In the phase 1 POLARIS trial, OriCAR-017 was administered to ten patients: nine in the dose-escalation phase (*n* = 3 each to 1 × 10^6^, 3 × 10^6^, and 6 × 10^6^ CAR-T cells per kg) and one in the dose-expansion phase (3 × 10^6^ CAR-T cells per kg) [52]. In total, 60% of patients had high-risk cytogenetics, 40% had baseline extramedullary lesions, and 80% had ISS stage II/III disease; median number of previous LOT was 5.5, and all patients were refractory to both proteasome inhibitors and immunomodulatory drugs. In total, 50% of patients had received prior anti-BCMA CAR-T therapy [52].

The most common grade 3/4 AEs were neutropenia, thrombocytopenia, leukopenia, and anemia (Table 3) [52]. On-target, off-tumor AEs related to GPRC5D were grade 1 nail disorders, grade 1/2 pruritus, and grade 2 dry skin. Nail disorders and dry skin resolved without intervention, and pruritus resolved with antipruritic treatment [52].

All patients experienced CRS events (all grade 1/2), which was grade 1 in nine (90%) patients [52]. All CRS cases were rapidly relieved with intervention with tocilizumab (*n* = 4), glucocorticoids (*n* = 1), or tocilizumab plus glucocorticoids (*n* = 3). Median time to CRS onset was 48 h, which progressively became shorter with increasing infusion doses, and median duration was 144 h (6 days). No neurological toxicities were reported. No dose-limiting toxicities were observed during the dose-escalation phase, and no deaths due to AEs were reported [52].

##### BMS-986393

The phase 1, multicenter, open-label trial of BMS-986393 enrolled 33 patients who received doses of 25 (*n* = 6), 75 (*n* = 9), 150 (*n* = 11), 300 (*n* = 6), and 450 (*n* = 1) × 10^6^ CAR-T cells [54]. In total, 49% (*n* = 16) had high-risk cytogenetics and 46% (*n* = 15) had baseline extramedullary lesions. In total, 55% (*n* = 18) had received prior BCMA-targeting therapies, 39% (*n* = 13) of whom received prior BCMA-targeting CAR-T therapy [54].

Treatment-emergent AEs were experienced by 29 patients (88%), which were grade 3/4 in 24 (73%) [54]. There were two dose-limiting toxicities in the groups that received 25 × 10^6^ and 75 × 10^6^ CAR-T cells: grade 4 neutropenia and/or thrombocytopenia. There were no deaths related to treatment [54].

The most common treatment-emergent AEs were predominantly hematologic: neutropenia, thrombocytopenia, and anemia [54] (Table 3). On-target, off-tumor AEs included skin-related AEs, dysgeusia/taste disorders, nail disorders, and dysphagia, which were all grade 1/2, and did not require management in most patients (79%). CRS was a commonly reported AE and was mostly grade 1/2. The median onset to CRS was day 3 and median duration was 4 days. Only two patients (6%) had ICANS, with both events being grade 1/2. A total of ten patients (30%) had headache. There were no cerebellar events [54].

#### *GPRC5D-related AEs and* management

A unique pattern of AEs has been reported in several trials of GPRC5D-targeting T-cell–redirecting therapies in MM (Table 3) [29, 42, 45, 52, 54]. Overall, most AEs were low grade and clinically manageable with appropriate dose modifications and supportive care. Given the novelty of these therapies, guidance on clinical management is required to ensure patients maintain quality of life and adherence to treatment. Consensus on the grouping of GPRC5D-specific AEs is required, given the differences observed across the five trials (Table 3).

Cross-trial comparisons are challenging in the context of the five reviewed agents, owing to differences in the grouping of some AEs and the differences in dosing for the bispecific antibodies as well as between CAR-T therapies and bispecific antibodies (a single infusion versus continuous dosing). Most skin-, nail-, and taste-related AEs were low grade, rarely required dose modifications, and were manageable [29, 41, 42, 45, 52, 54]. Rashes were responsive to emollients, and topical and oral corticosteroids in MonumenTAL-1, and topical glucocorticoids in the trial of MCARH109; in both trials, rashes did not result in study drug discontinuation [29, 41]. Supportive care for rash in MonumenTAL-1 enabled patients to continue to receive talquetamab without dose modification [41]. Most cases of dry mouth and dysphagia were grade 1/2 [29, 42, 45, 54]. Dysgeusia has a maximum severity of grade 2 per Common Terminology Criteria for Adverse Events guidelines (grade 2 defined as altered taste with change in diet [e.g., oral supplements]; noxious or unpleasant taste; loss of taste). Dysgeusia was a commonly reported AE but appeared to have a higher incidence with bispecific antibodies compared with CAR-T therapies [29, 42, 54]. Dysgeusia was managed with supportive care and, at times, dose adjustments [41, 42, 55]. It is unknown whether dysgeusia is a direct on-target, off-tumor effect because GPRC5D immunoreactivity in salivary glands is limited to resident plasma cells, and while GPRC5D is also expressed on filiform papillae, they are not responsible for taste [25, 41]. However, the larger class of family C metabotropic G-protein coupled receptors include taste receptors [56], which may explain the taste-associated AEs experienced with GPRC5D-targeting T-cell–redirecting therapies. Physicians should be mindful of the potential impact of oral toxicities on weight management and ensure that patients receive adequate nutritional support; weight-based medications, such as hypotensive and hypoglycemic drugs, may require dose adjustment. Regular dental check-ups should be encouraged. The impact of oral toxicities on quality of life has not been assessed, as commonly used questionnaires do not evaluate these toxicities; as such, assessing the impact of oral toxicities on quality of life is a question of high clinical interest. Nail-related AEs were reported frequently, were low grade, likely related to on-target activity, and while often resolved without intervention, were sometimes managed with emollients, nail hardeners, vitamin E oil, as well as hydration, biotin, and protective nail coverings based on investigator experience from the MonumenTAL-1 trial [29, 41, 42, 45, 52]. Differences in observed rates of GPRC5D-related AEs between bispecific antibodies and CAR-T therapies may be due to continuous dosing versus single-infusion dosing, different epitopes, or differences in drug distribution. Overall, the pattern of AEs did not always correspond with the location of keratinized tissue. Additional studies are ongoing to understand the underlying mechanisms involved in skin- and oral-related toxicities and to define management strategies for these AEs. Based on the experiences of patients treated with talquetamab, GPRC5D-targeting T-cell–redirecting therapies result in sustained clinically meaningful improvements in quality of life, despite the occurrence of these AEs throughout the course of treatment [57].

#### T-cell redirection–related AEs and management

CRS was commonly reported for all GPRC5D-targeting T-cell–redirecting therapies [29, 42, 45, 52, 54]. In trials of talquetamab and forimtamig, CRS mitigation strategies were incorporated into trial designs. For MonumenTAL-1, pre-treatment with a glucocorticoid, antihistamine, and paracetamol was required prior to all step-up doses and the initial full dose, as well as hospitalization for initial treatment doses [41, 42]. For forimtamig, CRS mitigation strategies included step-up dosing, use of corticosteroids pre-medication during cycle 1, and hospitalization for cycle 1 doses [28, 45]. Common supportive measures for CRS included tocilizumab, corticosteroids, and rarely vasopressors [29, 42, 45, 52]. ICANS occurred in approximately 10% of patients in both MonumenTAL-1 and the forimtamig trial [42, 45]; no patients in the OriCAR-017 trial had neurological toxicities. In the MCARH109 trial, one patient experienced ICANS and two patients had a grade 3 cerebellar disorder [29, 52]. Further investigation is required to better understand the presence of rare cerebellar events with CAR-T therapies, as they have not been reported with bispecific antibodies [29]; however, this observation may be due to differences in drug distribution between the two therapy types.

Infections have been reported with the use of T-cell–redirecting bispecific antibodies; however, infections were reported less frequently in trials of GPRC5D-targeting T-cell–redirecting bispecific antibodies compared with trials of BCMA- and Fc receptor-like protein 5–targeting bispecific antibodies [58–61], which may be due to the more limited expression profile of GPRC5D in the immune compartment compared with the expression profiles of these other target antigens [20, 21, 25–27]. Additionally, the lower expression of GPRC5D on normal plasma cells compared with malignant plasma cells may preserve normal plasma cells during treatment [21, 59]. This is underpinned by data showing that GPRC5D-targeting bispecific antibodies do not lead to a reduction in CD19 + B-cell levels over time based on experience with talquetamab [59]. Additionally, there was no decrease in IgG over time with talquetamab; this may mean that IV immunoglobulin is required less frequently with GPRC5D-targeting T-cell–redirecting bispecific antibodies compared with bispecific antibodies targeting other antigens [59].

#### Efficacy

##### Talquetamab

The key efficacy results of the MonumenTAL-1 trial, as well as from the trials of the other reviewed therapies, are summarized in Table 4 [42]. Overall response rate (ORR) was 74% (106/143) for 0.4 mg/kg QW and 73% (106/145) for 0.8 mg/kg Q2W. Median time to first confirmed response was 1.2 months for the 0.4 mg/kg QW cohort and 1.3 months for the 0.8 mg/kg Q2W cohort. Most responders had a very good partial response (VGPR) or better (59% and 57%, respectively). In patients who received prior T-cell–redirection therapies, ORR was 63% (72% in patients with prior CAR-T therapy; 44% in patients with prior bispecific antibody therapy). ORR in other key patient subgroups (0.4 mg/kg QW and 0.8 mg/kg Q2W, respectively) were 73% and 71% in triple-class refractory patients, and 71% in both groups in penta-drug refractory patients. ORR was consistent across other subgroups, including number of prior therapies, refractoriness to prior therapy, prior belantamab exposure, and baseline cytogenetic risk, except among patients with baseline plasmacytomas. Median duration of response was 9.3 and 13.0 months in the two cohorts, respectively, and 12.7 months in patients who received prior T-cell–redirection therapies [42].

**Table 4:** Efficacy and pharmacokinetics summary across GPRC5D-targeting agents.

	Talquetamab		Forimtamig^b^		MCARH109^c^ (*N* = 17)	OriCAR-017^d^ (*N* = 10)	BMS-986393^e^ (*N* = 19)
Patients, *n* (%)	0.4 mg/kg SC QW^a^ (*n* = 143)	0.8 mg/kg SC Q2W (*n* = 145)	IV cohort (*n* = 49)	SC cohort (*n* = 55)			
ORR	106 (74)	106 (73)	35 (71)	35 (64)	12 (71)	10 (100)	17 (90)
sCR	34 (24)	29 (20)	15 (31)	5 (9)	N/R	6 (60)	6 (32)
CR	14 (10)	18 (12)	2 (4)	9 (16)	N/R	0	3 (16)
≥CR	N/R	N/R	17 (35)	14 (26)	6 (35)	N/R	9 (47)
VGPR	37 (26)	36 (25)	12 (25)	15 (27)	N/R	4 (40)	1 (5.3)
≥VGPR	85 (59)	83 (57)	29 (59)	29 (53)	10 (59)	N/R	N/R
Median DOR^f^ (months)	9.3 (95% CI, 6.6–12.7)	13.0 (95% CI, 10.6–NE)	10.8 (range, 0.0–17.6)	12.5 (range, 1.2–12.5)	7.8 (95% CI, 5.7–NE)	N/R	N/R
Time to response							
Median time to first response, months^g^	1.2 (range, 0.2–10.9)	1.3 (range, 0.2–9.2)	1.4 (95% CI, 1.2–1.8)	1.6 (95% CI, 1.2–2.1)	N/R	N/R	N/R
Median time to best response, months^g^	2.2 (range, 0.8–12.7)	2.7 (range, 0.3–12.5)	N/R	N/R	N/R	3.1 (IQR, 2.0–5.1)	N/R

Data cut-off: September 12, 2022 (talquetamab); October 21, 2022 (forimtamig); June 30, 2022 (OriCAR-017); patients were enrolled between September 15, 2020, and June 16, 2021 (MCARH109); September 7, 2022 (BMS-986393).

*CI* confidence interval, *CR* complete response, *≥CR* complete response or better, *DOR* duration of response, *GPRC5D* G protein–coupled receptor class C group 5 member D, *IV* intravenous, *IQR* interquartile range, *NE* not estimable, *N/R* not reported, *ORR* overall response rate, *Q2W* every other week, *QW* weekly, *RP2D* recommended phase 2 dose, *SC* subcutaneous, *sCR* stringent complete response, *VGPR* very good partial response, *≥VGPR* very good partial response or better.

^a^In phase 1, 0.405 mg/kg SC QW was one of the two RP2Ds; 0.4 mg/kg SC QW was selected as final dosing concentration in phase 2 for operational convenience.

^b^Data presented are for all IV and SC doses combined.

^c^Three patients received 25 × 10^6^ CAR-T cells and 50 × 10^6^ CAR-T cells, six patients received 150 × 10^6^ CAR-T cells, and five patients received 450 × 10^6^ CAR-T cells.

^d^Three patients each received 1 × 10^6^ CAR-T cells per kg, 3 × 10^6^ CAR-T cells per kg, and 6 × 10^6^ CAR-T cells per kg, respectively, in the dose-escalation phase. In the dose-expansion phase, one additional patient received the RP2D of 3 × 10^6^ CAR-T cells per kg.

^e^Thirty-three patients received doses of BMS-986393 at 25 (*n* = 6), 75 (*n* = 9), 150 (*n* = 11), 300 (*n* = 6), and 450 (*n* = 1) ×10^6^ CAR-T cells.

^f^Median DOR not reached in both cohorts for talquetamab in those patients who achieved ≥CR.

^g^Calculated from *n* = 106 responders in each cohort of patients who received talquetamab.

##### Forimtamig

In the IV cohort, ORR was 71%, and 59% of patients achieved ≥VGPR; ORR in the SC cohort was 64%, with 53% of patients achieving a ≥VGPR [45]. Of the ten and 11 patients with prior anti-BCMA exposure in the IV and SC cohorts, respectively, five (50%) and six (55%) had a response. Median time to first response was 1.4 and 1.6 months in each cohort, respectively. Median DOR was 10.8 months in the IV cohort and 12.5 months in the SC cohort. At data cut-off, responses were still ongoing in 23/35 (66%) patients in the IV cohort and in 25/35 (71%) patients in the SC cohort [45].

##### MCARH109

In the overall population (*n* = 17), 71% of patients had a response [29]. Of these, 12 (71%) patients had a partial response (PR) or better, ten (59%) had VGPR or better, and six (35%) had a complete response (CR)/stringent CR (sCR) [29]. A ≥PR was observed in 7/10 patients (70%) who received prior BCMA-targeting therapies and received MCARH109 across all four dose levels, and in 3/6 patients (50%) treated at doses of 25 × 10^6^ to 150 × 10^6^ CAR-T cells [29].

##### OriCAR-017

In the overall population (*n* = 10), ORR was achieved by 100% of patients, including six patients (60%) with sCR and four patients (40%) with VGPR [52]. Median time to best response was 3.1 months and median time to ≥CR was 4.1 months. Of the five patients who relapsed following prior BCMA-targeting CAR-T therapy, two had sCR and three had VGPR [52].

##### BMS-986393

In the overall efficacy population (*N* = 19), 17 patients (90%) achieved an ORR [54]. Of these, six patients achieved sCR, three patients achieved a CR, and one patient achieved a VGPR [54].

#### Pharmacokinetics, pharmacodynamics, and immunogenicity

Data for talquetamab RP2Ds and forimtamig showed that both were associated with consistent T-cell activation and cytokine production, including interleukin-6 [42, 62]. Forimtamig also showed bone marrow infiltration and MM cell depletion early after treatment [62]. Preliminary data with forimtamig suggest that both SC and IV formulations led to similar safety and efficacy [45], although SC administration induced delayed and lower cytokine release compared with IV infusion [45]. In comparison, SC formulation of talquetamab, which is considered more convenient for patients and healthcare providers [63, 64], had a favorable pharmacokinetic (PK) profile compared with the IV formulation [42]. PK profiles were comparable across both talquetamab RP2Ds, with mean concentration-time profiles that were comparable between QW and Q2W dosing and maintained at or above the maximum EC_90_ values identified in an ex vivo cytotoxicity assay [42]. Anti-drug antibodies were detected in 20% of patients, which did not appear to impact safety, efficacy, or PK [42]. Immunogenicity data are not yet available with forimtamig.

CAR-T cell expansion was observed with MCARH109, OriCAR-017, and BMS-986393, which was dose dependent [29, 52, 54]. CAR-T cells were durable over time, with 90% of patients showing detectable OriCAR-017 at 1 month, 70% at 3 months, and 50% at 6 months [52]. Persistent MCARH109 was detected in the peripheral blood at 4 and 24 weeks in 100% of patients and at 52 weeks in 50% of patients [29]. BMS-986393 reduced soluble BCMA levels across all doses [54].

## Future directions

Additional trials are currently underway investigating the safety and efficacy of combining GPRC5D-targeting T-cell–redirecting agents with other anti-myeloma agents in patients with relapsed/refractory MM. These include trials evaluating the use of talquetamab in combination with teclistamab, daratumumab, pomalidomide, an anti-programmed cell-death protein-1 antibody, carfilzomib, and lenalidomide. MCARH109 is being evaluated in combination with MCARH125, a BCMA-targeting CAR-T therapy. The relatively low rates of high-grade cytopenia and severe infections, including infections leading to death, observed with these therapies and non-overlapping AE profiles with conventional MM agents may make GPRC5D-targeting bispecific antibodies versatile combination partners. With regard to the GPRC5D-associated AEs, further research is needed to assess the effectiveness of dose modification strategies, including reduced dose frequency and fixed duration dosing, as well as other mitigation measures, to more optimally balance on-target, off-tumor toxicity with the promising efficacy observed with these therapies. In addition to these trials, CAR–natural killer, bispecific–natural killer cell engager, and antibody-drug conjugate therapies may also offer advancements in treatment outcomes [65–68].

Additional trials are needed to determine the sequence in which GPRC5D-targeting T-cell–redirecting agents should be placed in the MM treatment paradigm. Based on the MonumenTAL-1 trial, efficacy outcomes in patients with prior T-cell redirection treatment were not as promising as in patients without prior T-cell redirection exposure; however, efficacy data with talquetamab in combination with either daratumumab or teclistamab in patients with prior exposure to T-cell redirection therapy is promising [31, 69]. Immune correlative analyses will be important to understand the impact of GPRC5D-targeting T-cell–redirecting therapies on immune system health, as will understanding mechanisms of resistance [33, 34]. As GPRC5D mRNA is expressed in samples from patients with smoldering MM, GPRC5D-targeting T-cell–redirecting therapies may provide clinical benefit in patients with pre-malignant disease, although the safety profile should be considered in relation to disease state and outcomes. Investigation into the use of GPRC5D-targeting T-cell–redirecting agents in the treatment of other plasma cell diseases is needed.

## Conclusions

GPRC5D is a validated target for MM therapies. In early phase trials, GPRC5D-targeting T-cell–redirecting agents have shown promising efficacy and manageable safety profiles, which need to be confirmed in phase 3 trials. GPRC5D-targeting T-cell–redirecting therapies, as monotherapy or in combination with other anti-myeloma agents, will expand the number of treatment options available for patients with MM. These therapies may provide options for patients who may need treatment with a novel mechanism of action that preserves BCMA-targeting therapy for later LOT, have experienced suboptimal response or antigen loss with other agents, or for patients who exhibit clonal heterogeneity [18, 26]. However, the optimal treatment sequence for patients with MM will need to be elucidated.

## Data Availability

The data sharing policy of Janssen Pharmaceutical Companies of Johnson & Johnson is available at https://www.janssen.com/clinical-trials/transparancy. As noted on this site, requests for access to the study data can be submitted through Yale Open Data Access (YODA) Project site at http://yoda.yale.edu.
