# Correction: GPRC5D as a novel target for the treatment of multiple myeloma: a narrative review

**DOI:** 10.1038/s41408-024-01018-6

**Published:** 2024-03-06

**Authors:** Paula Rodriguez-Otero, Niels W. C. J. van de Donk, Kodandaram Pillarisetti, Ingrid Cornax, Deeksha Vishwamitra, Kathleen Gray, Brandi Hilder, Jaszianne Tolbert, Thomas Renaud, Tara Masterson, Christoph Heuck, Colleen Kane, Raluca Verona, Philippe Moreau, Nizar Bahlis, Ajai Chari

**Affiliations:** 1 Clínica Universidad de Navarra, CCUN, University of Navarra, Pamplona, Spain; 2 Amsterdam University Medical Center, Vrije Universiteit Amsterdam, Amsterdam, The Netherlands; 3 Janssen Research & Development, Spring House, PA USA; 4 Janssen Research & Development, La Jolla, CA USA; 5 Janssen Scientific Affairs, Horsham, PA USA; 6 Janssen Research & Development, Raritan, NJ USA; 7 University Hospital Hôtel-Dieu, Nantes, France; 8 Arnie Charbonneau Cancer Research Institute, University of Calgary, Calgary, Alberta Canada; 9 Icahn School of Medicine at Mount Sinai, New York, NY USA

**Keywords:** Myeloma, Drug development, Immunotherapy

Correction to: *Blood Cancer Journal* 10.1038/s41408-023-00966-9, published online 02 February 2024

The graphical abstract was missing from this article and should have appeared as below.
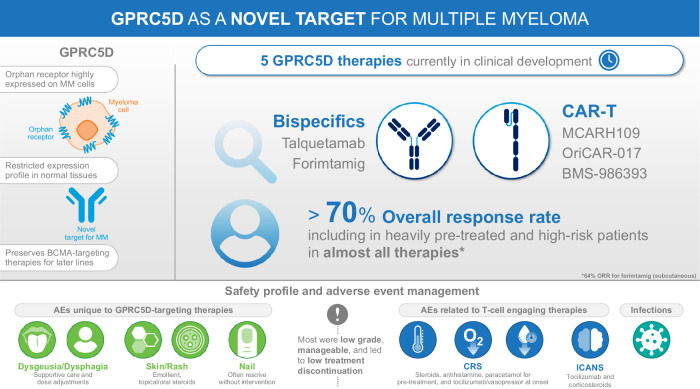


The original article has been corrected.

